# Involvement of the extracellular matrix proteins periostin and tenascin C in nasal polyp remodeling by regulating the expression of MMPs

**DOI:** 10.1002/clt2.12059

**Published:** 2021-09-06

**Authors:** Kun Du, Min Wang, Nan Zhang, Pei Yu, Ping Wang, Ying Li, Xiangdong Wang, Luo Zhang, Claus Bachert

**Affiliations:** ^1^ Department of Otorhinolaryngology Head and Neck Surgery Beijing Tongren Hospital Capital Medical University Beijing China; ^2^ Beijing Key laboratory of Nasal Diseases Beijing Institute of Otorhinolaryngology Beijing China; ^3^ Department of Oto‐Rhino‐Laryngology Upper Airways Research Laboratory Ghent University Hospital Ghent Belgium; ^4^ Department of Clinical Sciences, Intervention and Technology Division of ENT Diseases Karolinska Institute Stockholm Sweden

**Keywords:** MMPs, nasal polyps, periostin, remodeling, tenascin C, TIMPs, Matrix Metalloproteinase, Nasenpolyp, Periostal Protein, Gewebeumbau, Tenascin, Hemmer der Gewebe‐Metalloproteinase

## Abstract

**Background:**

Tissue remodeling caused by increased MMPs is involved in the pathogenesis of chronic rhinosinusitis with nasal polyposis (CRSwNP). We previously found higher levels of periostin and tenascin C in CRSwNPs, but whether they are associated with the dysregulation of MMPs is unknown. Therefore, the present study aimed to investigate the regulatory roles of these two ECM proteins in the expression of MMPs in nasal polyps.

**Methods:**

The concentrations of MMP‐2, MMP‐3, MMP‐7, MMP‐8, MMP‐9, MMP‐12, MMP‐13, TIMP‐1, TIMP‐2, TIMP‐3, TIMP‐4, periostin, and tenascin C in tissue homogenates of 51 patients with chronic rhinosinusitis with and without nasal polyps and 15 control subjects were measured and were analyzed by adjusted logistic regression and spearman correlation test. Primary human nasal polyp fibroblasts and epithelial cells were stimulated ex vivo with periostin and tenascin C and the gene expression of MMPs and TIMPs was determined by means of real‐time PCR.

**Results:**

The protein levels of MMP‐3, MMP‐7, MMP‐8, MMP‐9, TIMP‐1, TIMP‐2, periostin, and tenascin C were significantly higher in patients with CRSwNPs than in healthy control subjects. The adjusted logistic regression analyses showed that MMP‐3, MMP‐7, MMP‐8, MMP‐9, TIMP‐2, periostin, and tenascin C were related to the occurrence of CRSwNP. Spearman correlation test showed periostin was positively correlated with MMP‐3 and TIMP‐2, and tenascin C was positively correlated with MMP‐3, MMP‐7, MMP‐8, MMP‐9, and TIMP‐2. Periostin stimulated the gene expression of MMP‐3, MMP‐7, MMP‐8, and MMP‐9 in fibroblasts and MMP‐9 in epithelial cells ex vivo. Tenascin C stimulated the expression of MMP‐3, MMP‐7, MMP‐8, and MMP‐9 in epithelial cells. The expression of TIMPs in fibroblasts and epithelial cells was affected by neither periostin nor tenascin C.

**Conclusions:**

Periostin and tenascin C might be involved in the remodeling of nasal polyps by regulating the expression of different MMPs in epithelial cells and fibroblasts. Our findings have the potential to identify key factors of tissue remodeling in CRSwNPs.

## 1 BACKGROUND

Chronic rhinosinusitis (CRS) is a complex inflammatory disease in the upper airways characterized by 12 weeks of persistent symptoms involving nasal congestion, nasal discharge, facial pressure, loss of olfaction, cough, and fatigue. CRS affects approximately 11% of adults in Europe and about 12% of adults in the United States.^1^ Phenotypically, CRS is classified as CRS with nasal polyps (CRSwNP) and CRS without nasal polyps (CRSsNP), which represents about 20% and 80%, respectively, of patients with CRS.^2^ The prevalence of chronic rhinosinusitis with nasal polyps (CRSwNP) in Europe is estimated to be between 2.1% (France) and 4.4% (Finland) and is 4.2% in the United States.^1^ Compared with CRSsNP, CRSwNP manifests a higher disease severity and a higher risk of asthma comorbidity, and also showed a high risk for recurrence.^3^, ^4^ CRSwNP is characterized by the formation of edematous stroma and pseudocysts.^5^ Evidence shows that tissue remodeling is one of the main causes of the formation of nasal polyps.^6^

Tissue remodeling is a dynamic process which results in both extracellular matrix (ECM) production and degradation.^7^ Matrix metalloproteinase (MMPs) are zinc‐dependent and calcium‐dependent endopeptidases that are known to degrade and remodel ECM, which can be inhibited by the tissue inhibitors of metalloproteinase (TIMPs). Physiological remodeling in nasal mucosa plays an important role in wound repair, of which the histological features involve fibroblast proliferation, angiogenesis, and increased connective tissue formation.^7^, ^8^ Abnormal ECM production and degradation may contribute to pathological reconstruction with the formation of pathological tissue. For instance, excessive collagen deposition results in fibrosis in CRSsNP and higher levels of MMP‐induced tissue remodeling contribute to expansive histologic changes in CRSwNP.^9^

MMP‐induced tissue remodeling is strongly associated with ECM proteins, which play diverse roles and modulate cell‐matrix interactions to control cellular metabolism within the ECM.^10^ The ECM proteins are readily up‐regulated under pathological conditions.^11^ We previously found higher levels of ECM proteins, tenascin C and periostin, in NPs compared with controls.^12^ Periostin is a confirmed novel biomarker for the formation of nasal polyps and tenascin C is an indicator of inflammation.^10^, ^13^ However, whether the two ECM proteins contribute to the formation of CRSwNP via tissue remodeling is unknown. Previous studies have demonstrated higher levels of MMPs in NP tissues^9^, ^14^ and the association between the two ECM proteins (periostin and tenascin C) and the expression of MMPs beyond nasal mucosa.^15^, ^16^ Together with evidences of the expression of MMPs in fibroblasts and epithelial cells isolated from nasal mucosa^17^, ^18^ we hypothesized that periostin and tenascin C may be involved in CRSwNP remodeling by promoting MMPs expression in nasal fibroblasts and/or epithelial cells. To investigate this, we measured the concentrations of MMPs, TIMPs, periostin and tenascin C in tissue homogenates of patients with CRSsNP, CRSwNP and control subjects and analyzed their relationships; and stimulated ex vivo cultured nasal polyp‐derived primary human fibroblasts and epithelial cells with periostin and tenascin C to detect the gene expression of MMPs and TIMPs.

## 2 METHODS

### 2.1 Patients

The study enrolled 66 subjects from the Rhinology Department of Beijing Tongren Hospital affiliated with the Capital Medical University, including 14 patients with chronic rhinosinusitis without nasal polyposis (CRSsNP), 37 patients with CRSwNP, and 15 control subjects. The study was approved by the Ethics Committee of Beijing TongRen Hospital. Written informed consent was obtained from all subjects before enrollment in the study. The diagnosis of chronic rhinosinusitis (CRS) was in accordance with the European Position Paper on Rhinosinusitis and Nasal Polyps (EPOS2020).^19^ None of the study subjects had any history of malignancy, cystic fibrosis, ciliary dyskinesia, allergic fungal sinusitis, maxillary antrochoanal polyps, upper or lower respiratory tract infections within 2 weeks preoperatively, or autoimmune diseases. Subjects who had taken systemic or local glucocorticosteroids or antibiotics within the last 2 weeks were excluded from the study. Subjects undergoing rhinoseptoplasty because of anatomic variations were recruited as control subjects. Tissue samples were obtained from the inferior turbinates of the control subjects, the ethmoid mucosae of patients with CRSsNP, and the NPs of patients with CRSwNP. These samples were frozen and stored at −80°C until used for immunoassays.

### 2.2 Clinical data

A complete blood count was taken, and a computed tomography (CT) scan was performed for the enrolled patients. The percentage of eosinophils and neutrophils in circulating blood was determined using a Sysmex XE‐2100 Automated Hematology System (Sysmex). Based on a positive skin prick test, atopy to a panel of common allergens was confirmed: animal hair, tress, grasses, cereals, mugwort, dandelion, blaterlia germanica, pine, plantain, curvularia lunata, cardida albicans, penicillium notatum, alternaria tenuis, aspergillus fumigatus, giant ragweed, chenopodium album, humulus, black locust, dermatophagoides, derpmatophagoides. Images obtained from CT scans (Philips Healthcare) were scored according to the Lund‐Mackay scoring system (LMS).^20^

### 2.3 Tissue eosinophils and neutrophils counting

Samples of nasal tissues from patients with CRSwNP, CRSsNP and control were processed for histological evaluation by H&E stain. All stained samples were observed by two independent pathologists, who were blinded to the clinical diagnosis and characteristics of the patients. Eosinophils and neutrophils were assessed by bright‐field light microscopy (BX51, Olympus) at ×400 magnification. The counts were recorded as the mean of the counts for 10 non‐overlapping fields in the lamina propria.

### 2.4 Immunoassay

Tissue homogenates were prepared as previously described.^21^ Briefly, frozen nasal tissues were weighed and homogenized with an automated homogenizer (TissueLyser LT; Qiagen) for 2 min. The homogenates were then dissolved in 0.9% NaCl (1 ml of 0.9% NaCl per 0.1 g of tissue) with 1% protease inhibitor cocktail (Sigma‐Aldrich, St Louis, Mo) and centrifuged to collect the supernatants.

The prepared tissue homogenates were assayed for tenascin C (US Biological), periostin, MMP‐2, MMP‐3, MMP‐7, MMP‐8, MMP‐9, MMP‐12, MMP‐13 and TIMP‐1, TIMP‐2, TIMP‐3, and TIMP‐4 (R & D Systems) by commercially available kits.

### 2.5 Primary human nasal polyp fibroblast cell culture and ex vivo stimulations

Fibroblasts in nasal polyps were isolated based on a previously described method.^22^ Briefly, fresh nasal polyp samples were obtained from CRSwNP patients and were washed several times with phosphate‐buffered saline (PBS), supplemented with 200 U/mL penicillin and 200 U/mL streptomycin. Samples were diced and plated in 100‐mm tissue culture plates containing Hyclone RPMI 1640 medium with 10% fetal bovine serum (FBS; Gibco) and 100U/mL of penicillin and streptomycin. When a monolayer of fibroblast‐like cells was found to be confluent, the cells were digested and passaged. After three passages, the cells were stimulated with 1 ug/ml, 2 ug/ml, and 5 ug/ml of periostin or tenascin C for 24 h, with the culture medium used as the control. The collected cells were stored at −80°C for further gene expression detection. The cells were characterized by flow cytometry using anti‐human CD90‐FITC and anti‐human CD45‐PerCP antibodies (Miltenyi Biotech) as previously described.^23^ The purity was more than 96%.

### 2.6 Primary human nasal polyp epithelial cells culture and ex vivo stimulations

Nasal polyp epithelial cells were established according to a previously described method.^24^ Briefly, fresh nasal polyp samples were rinsed with PBS and digested in 1 mg/ml protease (protease from Streptomyces griseus, Type XIV; Sigma‐Aldrich) for 1 h at 37°C. Cell suspensions were centrifuged at 800 rpm for 5 min and resuspended in bronchial epithelial growth medium (BEGM, Lonza, Basel). Cells were plated for 1 h on 100‐mm tissue culture plates to remove contaminating fibroblasts. The isolated cells were seeded on rat‐tail collagen‐coated tissue culture plates at 37°C with 5% CO_2_. The culture medium was changed every other day until the cells reached confluence. The isolated nasal epithelial cells were trypsinised and seeded into 12‐well culture plates at a concentration of 5 × 10^5^ cells/mL in 1 ml BEGM with 10% FBS and 100 U/mL of penicillin and streptomycin. After reaching 80% confluence, the cells were stimulated with 1, 2, and 5 ug/ml of periostin or tenascin C for 24 h, with the culture medium used as the control. The collected cells were stored at −80°C for further gene expression detection.

### 2.7 Real‐time PCR

Total RNA from unstimulated and stimulated fibroblasts and epithelial cells was extracted using TRIzol reagent (Ambion‐Life Technologies). The RNA was reverse transcribed into first‐strand cDNA with random primer, and real‐time polymerase chain reaction (PCR) was subsequently performed using the ABI7500 PCR system (Applied Biosystems, Foster City, Calif). Primer sequences are given in Table S1. The PCR conditions were as follows: a 95°C denaturation step for 10 min followed by 40 cycles of 95°C denaturation (15 s) and 60°C annealing (1 min). Gene expression was normalized to the housekeeping gene β‐actin. The comparative cycle threshold (Delta Delta Ct) method was used for relative gene expression analysis.

### 2.8 Statistical analysis

All statistical data were analyzed using IBM SPSS Statistics, Version 21.0 (IBM Corp). A Kolmogorov–Smirnov test was used to analyze the data distribution and the Kruskal–Wallis test was used for multiple comparisons among the different groups. When the results were significantly different, the Mann‐Whitney *U* test was used for inter‐group comparison. Relationships between the various parameters were evaluated by Spearman correlation analysis. Univariate and adjusted logistic regression analyses were used to investigate the factors related with CRSwNP. The Wilcoxon matched pairs test was used to compare the gene expression of patients‐matched unstimulated and stimulated nasal polyp‐derived fibroblasts and epithelial cells.

## 3 RESULTS

### 3.1 Demographic and clinical characteristics

The clinical characteristics of the patients and controls are presented in the Table 1. There were no significant differences in the age, sex, smoker, asthma, aspirin‐exacerbated respiratory disease (AERD), and tissue and blood neutrophils among the three groups. The proportion of patients with atopy was significantly higher in patients with CRSsNP and CRSwNP than in controls. LMS scores and the levels of tissue and blood eosinophil (Eos) were significantly higher in patients with CRSsNP and CRSwNP than in controls. The counts of tissue Eos were significantly higher in patients with CRSwNP compared with CRSsNP.

**TABLE 1:** The clinical characteristics of the subjects

	Control	CRSsNP	CRSwNP	*p* value
	*N* = 15	*N* = 14	*N* = 37	
Age (y)	40.9 ± 12.6	44.4 ± 13.0	44.1 ± 13.4	0.695
Male, *n* (%)	8 (53.3)	3 (21.4)	9 (24.3)	0.056
Smoker, *n* (%)	4 (26.7)	3 (21.4)	15 (40.5)	0.357
Asthma, *n* (%)	0 (0)	1 (7.1)	4 (10.8)	0.409
Atopy, *n* (%)	0 (0)	4 (28.6)	15 (40.5)	0.014^a^ ^,^ ^b^
AERD	0/15	0/14	4/37	0.188
Tissue Eos (number/HPF)	0 (0–1)	6.5 (0–30)	45 (0–900)	0.001^a^ ^,^ ^b^ ^,^ ^c^
Tissue Neu(number/HPF)	0 (0–5)	0 (0–150)	0 (0–5)	0.754
Blood Eos (%)	1.6 (0.5–7.5)	4.9 (2.3–7.8)	4.8 (1–20.1)	0.008^a^ ^,^ ^b^
Blood Neu(%)	51.3 (39.1–59.4)	52.5 (40–60.5)	54 (34.5–69.4)	0.526
LMS	0 (0–2)	6 (2–16)	14 (2–24)	<0.001^a^ ^,^ ^b^ ^,^ ^c^

*Note*: Data are expressed as numbers (%), means ± standard deviation, or median (range). a, b, c were considered statistically significant (*p* < 0.05).

Abbreviations: AERD, aspirin‐exacerbated respiratory disease; Eos, eosinophil; HPF, high power field; LMS, Lund‐Mackay scoring system; Neu, neutrophil.

^a^ Control versus CRSsNP.

^b^ Control versus CRSwNP.

^c^ CRSsNP versus CRSwNP.

### 3.2 Expression of MMPs, TIMPs and ECM proteins in nasal tissue homogenates from patients with CRSsNP and CRSwNP

**FIGURE 1:**
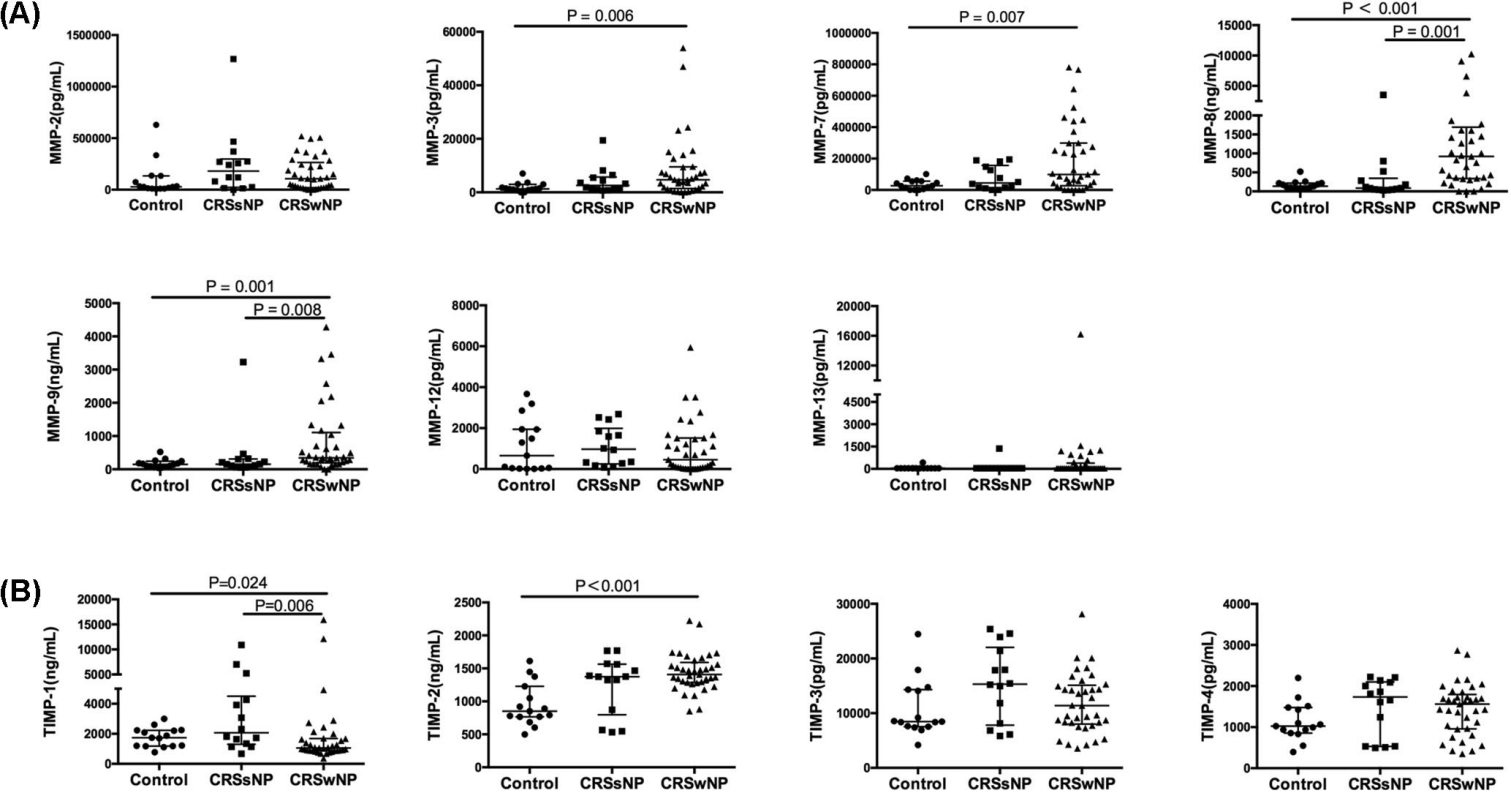
The protein levels of (A) Matrix metalloproteinases and (B) Tissue inhibitors of metalloproteinases in tissue homogenates from healthy control subjects and patients with CRSsNP and CRSwNP. Control subjects, *n* = 15; patients with CRSsNP, *n* = 14; patients with CRSwNP, *n* = 37. Data are presented as median and interquartile range. CRSsNP, chronic rhinosinusitis without nasal polyposis; CRSwNP, chronic rhinosinusitis with nasal polyposis

The protein levels of MMP‐2, MMP‐3, MMP‐7, MMP‐8, MMP‐9, MMP‐12, MMP‐13, TIMP‐1, TIMP‐2, TIMP‐3, TIMP‐4, periostin and tenascin C were examined in tissue homogenates from patients with CRS. Figure 1 shows the results. In comparison with healthy control subjects, no difference was found in patients with CRSsNP for all these MMPs and TIMPs, while increased levels of MMP‐3, MMP‐7, MMP‐8, MMP‐9 and TIMP‐1, and TIMP‐2 were found in patients with CRSwNP. The levels of MMP‐8 and MMP‐9 were higher in patients with CRSwNP than in patients with CRSsNP, but the level of TIMP‐1 was lower.

As significant differences for atopy, LMS and the levels of tissue and blood Eos were found among the three groups, we investigated whether the MMP levels were related with these parameters. No relationship between the atopy and MMPs was found (data not shown). We identified that tissue and blood eosinophilic inflammation was significantly related with the levels of MMP‐3 and MMP‐7 (Table 2). Additionally, significant correlations of LMS with MMP‐3, MMP‐7, MMP‐8, MMP‐9, TIMP‐1, and TIMP‐2 were found (Table 2).

**TABLE 2:** Correlations of MMPs and TIMPS in nasal tissue homogenates with eosinophilic inflammation and nasal disease severity from all the enrolled subjects

	Tissue Eos					
	(Number/HPF)		Blood Eos (%)		LMS	
	*r* value	*p* value	*r* value	*p* value	*r* value	*p* value
MMP‐3	0.366	0.008	0.451	<0.001	0.263	0.031
MMP‐7	0.418	0.002	0.260	0.033	0.306	0.012
MMP‐8	0.117	0.413	0.049	0.696	0.400	0.001
MMP‐9	0.164	0.251	0.176	0.155	0.345	0.004
TIMP‐1	−0.006	0.964	−0.006	−0.962	−0.344	0.004
TIMP‐2	−0.01	0.944	−0.034	0.788	0.290	0.017

Abbreviations: LMS, Lund‐Mackay scoring system; MMP, matrix metalloproteinases; TIMP, tissue inhibitors of metalloproteinase.

**FIGURE 2:**
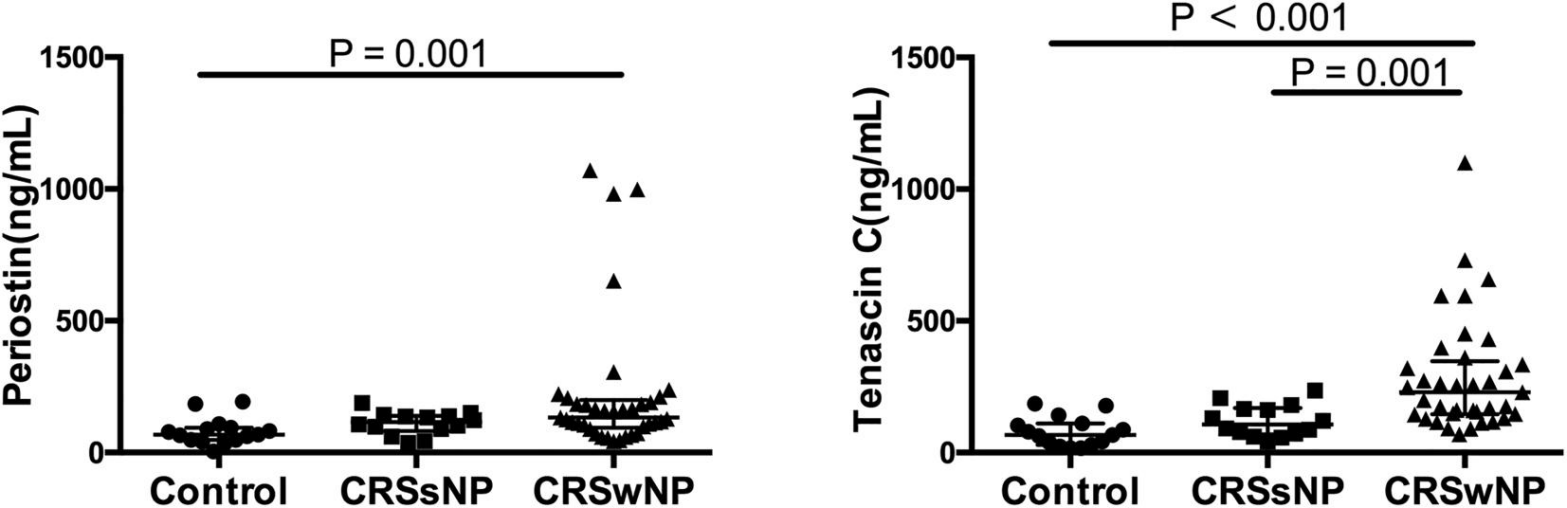
The protein levels of the extracellular matrix proteins, periostin and tenascin C, in tissue homogenates from healthy control subjects and patients with CRSsNP and CRSwNP. Control subjects, *n* = 15; patients with CRSsNP, *n* = 14; patients with CRSwNP, *n* = 37. Data are presented as median and interquartile range. CRSsNP, chronic rhinosinusitis without nasal polyposis; CRSwNP, chronic rhinosinusitis with nasal polyposis

In comparison to healthy control subjects, both periostin and tenascin C were up‐regulated in patients with CRSwNP, but not in patients with CRSsNP (Figure 2). Additionally, a significant higher levels of tenascin C were found in patients with CRSwNP versus patients with CRSsNP.

### 3.3 The association of MMPs, TIMPs, and ECM proteins with CRSwNP

Further, we investigated whether the levels of MMPs, TIMPs, and ECM proteins that showed significant differences between CRSwNPs, CRSsNPs and controls, were associated with the formation of nasal polyps. Using univariate logistic regression analysis, we found that MMP‐3, MMP‐7, MMP‐8, MMP‐9, TIMP‐2, periostin, and tenascin C were associated with CRSwNPs (Table 3). When the analysis was adjusted for CRSsNP, the odds ratios (OR) were 1.391, 1.015, 1.005, 1.006, 1.006, 1.013, and 1.029 for MMP‐3, MMP‐7, MMP‐8, MMP‐9, TIMP‐2, periostin, and tenascin C, respectively (*p* < 0.05).

**TABLE 3:** Univariate and adjusted logistic regression analyses of CRSwNP

CRSwNP versus Non‐CRSwNP				
	Univariate		Adjusted for CRSsNP	
Variable	OR (95% CI)	*p* value	OR (95% CI)	*p* value
MMP‐3 (ng/mL)	1.142 (1.004–1.299)	0.043	1.391 (1.035–1.870)	0.029
MMP‐7 (ng/mL)	1.008 (1.002–1.015)	0.007	1.015 (1.001–1.028)	0.033
MMP‐8 (ng/mL)	1.002 (1.001–1.003)	0.005	1.005 (1.001–1.009)	0.014
MMP‐9 (ug/mL)	1.006 (1.001–1.010)	0.007	1.006 (1.001–1.011)	0.028
TIMP‐1 (ug/mL)	0.931 (0.772‐1.122)	0.451	NA	NA
TIMP‐2 (ng/mL)	1.003 (1.001–1.005)	0.001	1.006 (1.003–1.009)	<0.001
Periostin (ug/mL)	1.003 (1.001–1.005)	0.001	1.013 (1.003–1.024)	0.011
Tenascin C (ug/mL)	1.020 (1.009–1.030)	<0.001	1.029 (1.011–1.048)	0.002

Abbreviations: MMP, matrix metalloproteinases; TIMP, tissue inhibitors of metalloproteinase.

### 3.4 Correlations between MMPs and TIMPs and ECM proteins

Next, we investigated the correlations between these MMPs and TIMPs and the ECM proteins. As shown in Table 4, periostin was positively correlated with MMP‐3 and TIMP‐2, and tenascin C was positively correlated with MMP‐3, MMP‐7, MMP‐8, MMP‐9 and TIMP‐2 in all subjects. Moreover, periostin tended to be positively correlated with MMP‐9 with a *p* value of 0.053.

**TABLE 4:** Correlations of the protein levels of periostin and tenascin C with MMPs and TIMPs in nasal tissue homogenates from all subjects

	Periostin		Tenascin C	
	*r* value	*p* value	*r* value	*p* value
MMP‐3	0.334	0.006	0.502	<0.001
MMP‐7	0.192	0.192	0.318	0.009
MMP‐8	0.222	0.222	0.438	<0.001
MMP‐9	0.239	0.053	0.425	<0.001
TIMP‐1	−0.13	−0.13	−0.132	0.289
TIMP‐2	0.251	0.042	0.453	<0.001

Abbreviations: MMP, matrix metalloproteinases; TIMP, tissue inhibitors of metalloproteinase.

### 3.5 Effect of periostin and tenascin C treatment on the gene expression of MMPs and TIMPs in nasal polyp‐derived primary fibroblasts ex vivo

**FIGURE 3:**
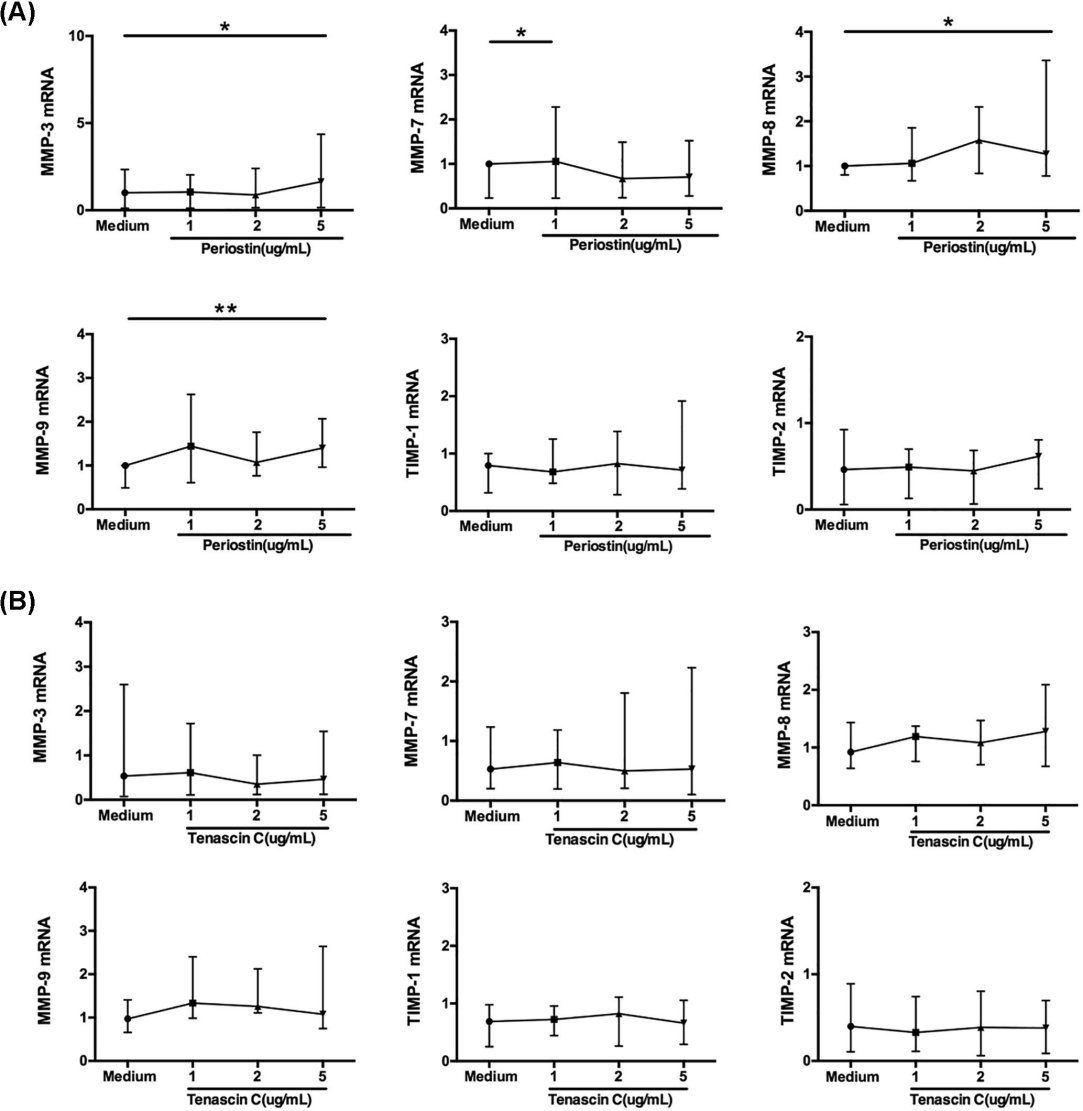
Gene expression of MMPs and TIMPs in primary fibroblasts from nasal polyp tissue stimulated by (A) periostin and (B) tenascin C. *N* = 10. The statistical significance of differences in expression was assessed by means of the Wilcoxon matched‐pairs test. Data are presented as median and interquartile range. *p < 0.05, **p < 0.01. MMP, matrix metalloproteinases; TIMP, tissue inhibitors of metalloproteinase

Regarding the correlations of ECM proteins with the expression of MMPs and TIMPs, and the fact that fibroblasts are the main source of MMPs and TIMPs, we first observed the regulation by ECM proteins of MMP‐3, MMP‐7, MMP‐8, MMP‐9 and TIMP‐1, and TIMP‐2 gene expression in nasal polyp‐derived fibroblasts. The results showed that periostin (1 ug/mL) significantly increased the expression of MMP‐7 and 5 ug/mL periostin stimulation significantly increased the expression of MMP‐3, MMP‐8 and MMP‐9, but had no effect on the expression of TIMP‐1 and TIMP‐2 (Figure 3). However, tenascin C stimulation did not change the expression of any of these MMPs and TIMPs at the gene level in the fibroblasts (Figure 3).

### 3.6 Effect of periostin and tenascin C treatment on the gene expression of MMPs and TIMPs in nasal polyp‐derived primary epithelial cells ex vivo

**FIGURE 4:**
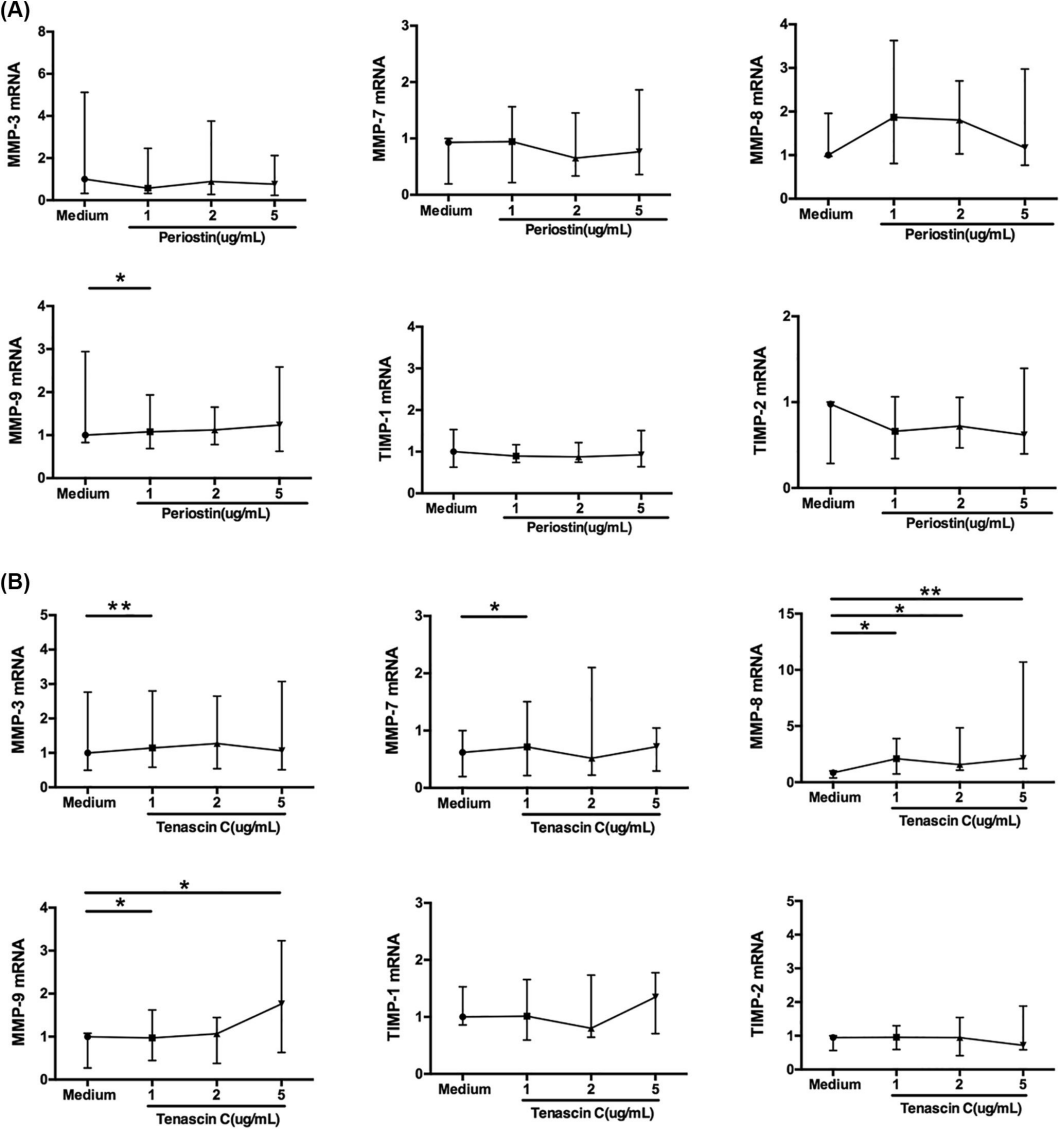
Gene expression of MMPs and TIMPs in primary epithelial cells from nasal polyp tissue stimulated by (A) periostin and (B) tenascin C. *N* = 11. The statistical significance of differences in expression was assessed by means of the Wilcoxon matched‐pairs test. Data are presented as median and interquartile range. *p < 0.05, **p < 0.01. MMP, matrix metalloproteinases; TIMP, tissue inhibitors of metalloproteinase

As epithelial cells are a source of MMPs and TIMPs, we next observed the regulation by ECM proteins of the expression of those MMPs and TIMPs in nasal polyp‐derived epithelial cells. Contrary to the results in the fibroblasts, periostin treatment in the epithelial cells had almost no effect on the expression of those MMPs and TIMPs, except for the up‐regulation of MMP‐9 when stimulated with 1ug/mL of periostin (Figure 4); while tenascin C stimulation (1 ug/mL) significantly increased the expression of MMP‐3, MMP‐7, MMP‐8 and MMP‐9 (Figure 4). Additionally, 5 ug/mL of tenascin C treatment significantly increased the expression of MMP‐8 and MMP‐9 markedly stronger.

## 4 DISCUSSION

Tissue remodeling is a cardinal CRSwNP pathogenesis, which is a dynamic process of ECM formation and degradation, leading to changes in tissue architecture. MMPs are key enzymes with proteolytic activities that can degrade various ECM components and can lead to progressive histologic changes. Anti‐MMP treatment has been shown to modify polyp size and improve postoperative healing outcome.^25^ Further investigation is needed of the mechanism by which MMPs are stimulated in polyp tissues. In this context, our results helped to address the knowledge gap by analyzing the correlation of ECM proteins with MMPs and TIMPs in patients, and by ex vivo experiments. We identified that there is a positive correlation of periostin and tenascin C with MMPs and found that periostin and tenascin C could stimulate the ex vivo expression of MMPs mainly in different cells derived from nasal polyps, periostin in fibroblasts and tenascin C in epithelial cells.

Previous studies provided evidence for higher levels of MMP‐1, MMP‐2, MMP‐3, MMP‐7, MMP‐8, and MMP‐9 in nasal polyps.^14^, ^26^, ^27^ Correspondingly, we found that MMP‐3, MMP‐7, MMP‐8 and MMP‐9 were increased in CRSwNPs compared with controls, and MMP‐8 and MMP‐9 were increased in CRSwNPs compared with CRSsNP. Higher levels of MMPs within nasal polyps may result in a more severe tissue remodeling process and subsequent expansive histologic changes of nasal polyps. We identified the correlation of MMP‐3 and MMP‐7 with tissue and blood eosinophilic inflammation. We also demonstrated that the levels of MMP‐3, MMP‐7, MMP‐8, and MMP‐9 were related with the disease severity of CRS. We failed to observe a difference in MMPs between CRSsNP and the controls. Our results verified the roles of MMPs in the pathogenesis in CRSwNP patients, but not in CRSsNP patients.

We also found higher levels of periostin in nasal polyps compared with the controls and higher levels of tenascin C in nasal polyps compared with CRSsNP and the controls. Thus, we investigated whether there exists a relationship between the two ECM proteins and MMPs. The results showed that the ECM proteins were positively related to MMP‐2, MMP‐3, MMP‐7, MMP‐8 and MMP‐9. In this context, we speculated that periostin and tenascin C might be able to regulate the expression of these MMPs within nasal polyps. Fibroblasts and the nasal epithelium are both sources of MMPs in NPs.^18^, ^28^ Additionally, periostin and tenascin C have been reported to stimulate the expression of MMPs beyond nasal polyps.^15^, ^29^, ^30^ Thus, we investigated the roles of periostin and tenascin C in promoting the expression of MMPs via fibroblasts and the nasal epithelium in nasal polyps. Our results demonstrated that periostin could stimulate the expression of MMPs via fibroblasts, while tenascin C worked mainly via epithelial cells. The up‐regulated MMPs stimulated by periostin and tenascin C were in correspondence with the MMPs that showed significantly higher levels in tissue homogenates of CRSwNP. The ex vivo findings suggest that periostin and tenascin C may contribute to the formation of NPs via regulating the expression of MMPs in different cells.

Previous studies have found evidence that periostin can stimulate the expression of MMPs. Mukundan et al.^30^ found that periostin induced the expression of MMP‐13 in cartilage and Hakuno et al.^15^ revealed that periostin could promote the levels of MMP‐2 and MMP‐13 from valvular interstitial cells, and could increase the expression of MMP‐2 and MMP‐9 in macrophages in vitro. However, within nasal polyps, if and how periostin induces the expression of MMPs is still unknown. We showed that periostin induced the expression of MMP‐3, MMP‐7, MMP‐8, and MMP‐9 in nasal fibroblasts and the expression of MMP‐9 in nasal epithelial cells. Higher levels of MMP‐9 indicated an unfavorable outcome of CRSwNPs after endoscopic sinus surgery (ESS).^31^, ^32^ In this study, we found higher levels of MMP‐9 in CRSwNP versus CRSsNP and identified that periostin induced the expression of MMP‐9 in fibroblasts, suggesting that periostin might be able to mediate, at least in part, the pathogenesis of CRSwNP via regulating the production of MMP‐9 in nasal fibroblasts.

Tenascin C, as an ECM protein, could be increased in parallel with MMPs in some pathological states.^16^ However, the regulatory role of tenascin C on MMPs in nasal polyps remains unknown. In the present study, we found that tenascin C induced the expression of MMP‐3, MMP‐7, MMP‐8, and MMP‐9 in the nasal epithelium. There is substantial evidence that tenascin C contributes to tissue remodeling via the upregulation of MMP‐9 in the mouse model of the cardiovascular system (e.g., cardiac remodeling, hepatic ischemia/reperfusion, subarachnoid hemorrhage, etc.).^16^, ^29^, ^33^, ^34^ Recently, Kanagala et al.^35^ found higher levels of plasma MMP‐8 in heart failure patients with tenascin C above the median plasma concentration than in those with tenascin C below the median plasma concentration. Consistent with this, we found a positive correlation of tenascin C with MMP‐8 in nasal tissue homogenates in this study and observed that tenascin C could dramatically promote the expression of MMP‐8 in nasal polyp epithelial cells, indicating tenascin C might be the main factor driving the expression of MMP‐8. Recently, the pro‐inflammatory role of MMP‐8 via isolated macrophages was identified in mice.^36^ Our data addresses the knowledge gap and suggests that besides tissue remodeling, tenascin C has the potential to enhance the inflammation within nasal polyps. This hypothesis needs to be further examined in the future.

Besides tissue remodeling, there are many other pathogenic mechanisms underlying CRSwNP, involving epithelial dysfunction, angiogenesis, imbalanced immunological response, etc.^5^, ^37^ Of note, MMP‐induced tissue remodeling is a downstream event of epithelial dysfunction and type 2 inflammation.^5^ For instance, during epithelial impairment, epithelial‐to‐mesenchymal transition (EMT) occurs and MMPs (MMP‐2 and MMP‐9) would be produced by myofibroblasts, which expands the size of the basement membrane and induces epithelial remodeling. Additionally, the type 2 inflammation molecule, IL‐13, promotes the differentiation of monocytes to become M2 and contributes to subsequent fibrin crosslinking. Based on the ongoing remodeling process, MMP‐induced expanded change may convert the nasal tissues to polyps. Recently, an in vivo animal study found IgE also could induce remodeling via fibroblasts and anti‐IgE treatment inhibited the process.^38^ It indicates that targeting the upstream signaling could control tissue remodeling. However, which factors regulates MMP‐induce remodeling within nasal polyps is still unclear. We previously found that IL‐4 and IL‐13 could induce periostin expression in nasal polyps.^12^ In the present study, we verified that periostin might promote tissue remodeling via inducing the expression of MMPs. Our results indicates that anti‐IL‐4/IL‐13 treatment might have utility for controlling the remodeling process within nasal polyps, which has been confirmed to effectively treat severe CRSwNPs.^37^ Given the roles of periostin and tenascin C in promoting the expression of MMPs in CRSwNPs, our results indicate that anti‐periostin or anti‐tenascin C treatment might be able to inhibit tissue remodeling within nasal polyps, which still needs to be further verified by in vivo animal studies.

To our knowledge, this is the first study to describe the role of periostin and tenascin C in regulating the expression of MMPs in NP. The degradation of ECM in polyp tissues induced by MMPs is a risk factor for the morbidity of CRSwNP.^5^, ^13^ In addition, the mechanisms by which MMPs are synthesized need to be further investigated. We revealed that fibroblasts and the nasal epithelium are both sources of MMPs within nasal polyps. TIMP‐1 might be able to reduce the effects of the expression of MMP‐9; however, the levels of MMP‐9 and TIMP‐1 were negatively correlated with disease severity in CRS.^39^, ^40^ Although higher levels of TIMP‐1 and TIMP‐2 were found in CRSwNPs, we failed to verify the regulatory roles of periostin and tenascin C on TIMPs. In this regard, further studies are required to illuminate how TIMPs are induced in nasal polyps. It has been known that CRSwNP patients prominently manifest eosinophilic inflammation^5^ and periostin has been reported to have a role in orchestrating eosinophil infiltration.^41^, ^42^ Our data provide new insight into the role of periostin in the pathogenesis of CRSwNP, which promotes tissue remodeling via MMP production by fibroblasts during inflammation. Tenascin C contributes to tissue remodeling in a different manner. Tenascin C stimulates the production of MMPs mainly in the nasal epithelium, not in fibroblasts, and it mainly stimulates the production of MMP‐8. Among the up‐regulated MMPs in this study, MMP‐9 is extensively confirmed to be increased in NPs.^43^, ^44^ We showed that periostin and tenascin C are both able to stimulate the expression of MMP‐9 via fibroblasts and the nasal epithelium. There are several limitations to our study. First, the assessment of the tissue remodeling molecules was based only on the mRNA expression, which needs to be verified according to the levels of corresponding proteins in NP tissues. Second, the number of cases involved in the ex vivo experiments is relatively small. Third, we failed to study the role of periostin and tenascin C in fibroblasts and nasal epithelial cells in controls and CRSsNP, which could help to prove whether the remodeling ex vivo was observed only in CRSwNPs. Finally, we failed to investigate the regulatory role of periostin and tenascin C on patients with different endotypes, including eosinophilic and non‐eosinophilic CRSwNPs, which need further investigation.

## 5 CONCLUSIONS

Our study showed the correlation of periostin and tenascin C with some members of the MMP family, which were confirmed to be increased in patients with CRSwNP and identify that both periostin and tenascin C have regulatory roles in the expression of these MMP, acting on different types of cells, mainly fibroblasts for periostin and nasal epithelium for tenascin C. We provide evidence for the pathogenic roles of periostin and tenascin C in the formation of nasal polyps. Our findings have the potential to identify key factors enhancing tissue remodeling, which will be necessary to further uncover the pathogenesis of CRSwNPs.

## CONFLICT OF INTERESTS

No author has any competing interests to declare.

## AUTHOR CONTRIBUTIONS

Min Wang and Kun Du conceived and designed the experiments. Kun Du and Min Wang wrote the manuscript. Kun Du, Min Wang, Ping Wang, Pei Yu, and Ying Li performed the experiments. Nan Zhang, Xiangdong Wang, Luo Zhang, and Claus Bachert revised the manuscript.

## CONSENT FOR PUBLICATION

Not applicable.

## INFORMATION ON THE ADDITIONAL FILE

Additional file 1 is a word document (.doc), of which the title of data is Primers used for quantitative RT‐PCR analysis. It showed the information of primers used in the present study.

## Supporting information

Supporting Information S1
