# Supplementary material for: Involvement of the extracellular matrix proteins periostin and tenascin C in nasal polyp remodeling by regulating the expression of MMPs
Source: Clin Transl Allergy. 2021 Sep 6;11(7):e12059. doi: 10.1002/clt2.12059 (PMC8420995; doi:10.1002/clt2.12059)
Supplement: Supplementary file 1 — Supporting Information S1 [file CLT2-11-e12059-s001.docx]

| **Table S1** Primers used for quantitative RT-PCR analysis. | | |
| --- | --- | --- |
| Primer | Sequence | AT (°C) |
| MMP-3 | (F) 5’-AGGCTGTATGAAGGAGAGGCTGAT-3’ | 60 |
|  | (R) 5’-AGTGTTGGCTGAGTGAAAGAGACC-3’ |  |
| MMP-7 | (F) 5’-TGTATGGGGAACTGCTGACA-3’ | 60 |
|  | (R) 5’-GCGTTCATCCTCATCGAAGT-3’ |  |
| MMP-8 | (F) 5’-CCTTGCTAAGGACTACTGGGC-3’ | 60 |
|  | (R) 5’-CTGGCCCATTTGGGTTTGGA -3’ |  |
| MMP-9 | (F) 5’-GCCGACTTTTGTGGTCTTCC-3’ | 60 |
|  | (R) 5’-TACAAGTATGCCTCTGCCAGC-3’ |  |
| TIMP-1 | (F) 5’-CTGTTGTTGCTGTGGCTGAT-3’ | 60 |
|  | (R) 5’-ACTTGGCCCTGATGACGAG-3’ |  |
| TIMP-2 | (F) 5’-CGTTTTGCAATGCAGATGTA-3’ | 60 |
|  | (R) 5’-TCCTCTTGATAGGGTTGCCA-3’ |  |
| β-actin | (F) 5’-GATCCACATCTGCTGGAAGG-3’ | 60 |
|  | (R) 5’-AAGTGTGACGTTGACATCCG-3’ |  |
| At, annealing temperature | | |

**Online supplementary materials**
